# Examining Measures of Weight as Risk Factors for Sport-Related Injury in Adolescents

**DOI:** 10.1155/2016/7316947

**Published:** 2016-07-20

**Authors:** Sarah A. Richmond, Alberto Nettel-Aguirre, Patricia K. Doyle-Baker, Alison Macpherson, Carolyn A. Emery

**Affiliations:** ^1^Child Health Evaluative Sciences, Hospital for Sick Children, 555 University Avenue, Toronto, ON, Canada M5G 1X8; ^2^Sport Injury Prevention Research Centre, Faculty of Kinesiology, University of Calgary, 2500 University Drive, Calgary, AB, Canada T2N 1N4; ^3^School of Kinesiology and Health Science, York University, 4700 Keele Street, Toronto, ON, Canada M3J 1P3; ^4^Alberta Children's Hospital, Institute for Child and Maternal Health, 2888 Shaganappi Trail NW, Calgary, AB, Canada T3B 6A8; ^5^Departments of Pediatrics and Community Health Sciences, Cumming School of Medicine, University of Calgary, 2500 University Drive, Calgary, AB, Canada T2N 1N4

## Abstract

*Objectives*. To examine body mass index (BMI) and waist circumference (WC) as risk factors for sport injury in adolescents.* Design*. A secondary analysis of prospectively collected data from a pilot cluster randomized controlled trial.* Methods*. Adolescents (*n* = 1,040) at the ages of 11–15 years from two Calgary junior high schools were included. BMI (kg/m^2^) and WC (cm) were measured from direct measures at baseline assessment. Categories (overweight/obese) were created using validated international (BMI) and national (WC) cut-off points. A Poisson regression analysis controlling for relevant covariates (sex, previous injury, sport participation, intervention group, and aerobic fitness level) estimated the risk of sport injury [incidence rate ratios (IRR) with 95% confidence intervals (CI)].* Results*. There was an increased risk of time loss injury (IRR = 2.82, 95% CI: 1.01–8.04) and knee injury (IRR = 2.07, 95% CI: 1.00–6.94) in adolescents that were overweight/obese; however, increases in injury risk for all injury and lower extremity injury were not statistically significant. Estimates suggested a greater risk of time loss injury [IRR = 1.63 (95% CI: 0.93–2.47)] in adolescents with high measures of WC.* Conclusions*. There is an increased risk of time loss injury and knee injury in overweight/obese adolescents. Sport injury prevention training programs should include strategies that target all known risk factors for injury.

## 1. Introduction

Sport injury is the leading cause of injury in Canadian youth [1]. In the 2009-2010 Canadian Community Health Survey, children over the age of 12 reported sport as the activity where their most serious injury occurred, a total of 1,470,000 injuries over a one-year period [2]. Sport injury represents a large burden on both direct and indirect health care costs, including rehabilitation for the injury itself, and the costs associated with children spending time away from school and subsequent sport and physical activity. Lifelong participation in sport and physical activity is particularly important, as overweight/obesity is an established epidemic [3]. Overweight/obesity in youth in Canada has increased in past decades; the prevalence of obesity in 2004 was 2.5 times higher than measures in the late 1970s [3]. Self-report body mass index (BMI) data collected in the 2013 Health Indicator Profile reported that 421,350 Canadian children at the ages of 12–17 years were overweight or obese [3].

As part of a public health approach to reducing an injury problem, understanding the risk factors that place a child at increased risk for sport injury is a key part of the prevention pathway. Pediatric sport injury literature has long established nonmodifiable risk factors for sport injury to include age, sex, and previous injury, and there is some evidence for modifiable risk factors including low fitness levels (e.g., poor aerobic fitness and lack of preseason training) [4]. What is lesser known is the association of measures of obesity as independent risk factors for increases in sport injury rates in youth. This is not due to the lack of investigation, but rather a lack of consensus between studies, most likely due to differing methods of assessment of body composition, populations (sport versus non-sport specific), injury definitions, and study design, reducing the ability to synthesize data across studies. There are studies that demonstrate that children with higher BMI measures are at increased risk of a sport-related injury including Nilstad et al. (2014) where multivariable analyses demonstrated that female soccer players with a higher BMI were associated with greater odds of lower extremity injury (OR = 1.51, 95% CI: 1.21–1.90) [5]. There are also studies that demonstrate a protective effect for injury in children with higher BMI measures including Ezzat et al. (2014) who demonstrate a statistically significant reduction in the odds of sport injury in obese adolescents compared to their healthy weight counterparts (OR = 0.72, 95% CI: 0.53–0.99) [6]. The mixed results demonstrated in these studies may be due to the inconsistent measure and categorization of healthy weight in children, in addition to the lack of prospective data collection and direct measures of weight and height.

The current study is a follow-up to a previously reported study examining BMI as a risk factor for sport injury in adolescent youth in Alberta, Canada [7]. This study found that there was an increased odds of sport injury in obese adolescents compared to healthy weight adolescents; however, recommendations for future research included using prospective study designs and direct measures of weight [7]. Therefore, the aim of this study was to examine direct measures of BMI in youth and the association of these measures with sport-related injury.

## 2. Materials and Methods

This was a secondary analysis of data collected from a prospective, pilot cluster randomized controlled trial (RCT) in Calgary, Alberta, Canada [8]. The pilot study examined the effectiveness of a sport injury prevention program in junior high youth, comparing an intervention program implemented in physical education classes in two junior high schools (one intervention school and one control school) [8]. The two junior high schools chosen for this study were selected from similar, socioeconomic demographics. Students and their parent/guardian signed the consent form, approved by the Office of Medical Bioethics, University of Calgary, and the Calgary Board of Education.

The main outcome measure in this study was sport injury, defined as any injury resulting from sporting activity that required medical attention, time loss, and/or removal of the participant from the sporting activity. Secondary injury definitions included lower extremity injury (injury of the hip, knee, ankle, lower leg, and foot), time loss injury (an injury that resulted in time away from sport, physical activity, or school, greater than 7 days), and knee and ankle injury. Students reported any injury sustained in a sporting activity to the physical education teacher who instructed and assisted the student in completing an injury report form. Injury report forms were initiated by the student and followed up by a study physiotherapist who assessed each reported injury. The injury report forms used in this study have been previously validated in a sporting youth population [9, 10].

A certified exercise physiologist collected all weight and fitness measures (BMI, waist circumference, aerobic fitness, and vertical jump) and reliability was demonstrated using a one-week test-retest protocol [8]. BMI (kg/m^2^) was estimated from direct measures of height (cm) and weight (kg) and overweight and obese categories were created using validated age and sex specific, international cut-off points [11]. The overweight and obese categories were combined to create one variable in this study. Included in the healthy categorization were children with low BMI measures. The health effects of cut-off points corresponding to low BMI measures in children have not been validated as markers of disease risk, particularly with injury [11].

Waist circumference (WC) was collected at baseline and measured to the nearest 0.50 cm using anthropometric tape. The protocol for all physical measures was taken from the Canadian Physical Activity, Fitness and Lifestyle Appraisal [12]. Waist circumference was then categorized into a binary variable (healthy or unhealthy) using national reference data [13].

Participating students from both schools completed a baseline medical questionnaire (including questions about previous six-week and one-year injury history) and weekly sport participation journal (including the total sport participation hours over the study period). Previous one-year injury as a binary variable (yes, no) was included in the final model. Sport participation was collected prospectively from a weekly sport participation journal, completed in the student's health classes. The total number of hours of sport participation was used as the offset in a Poisson regression model, controlling for the amount of play between students. Indirect aerobic fitness was collected using the Leger 20-metre shuttle run and used as a covariate in the model [14]. The child's maximum stage recorded was converted into a VO_2_ measure, represented as the volume of oxygen consumption in millimetres per kilogram of body weight per minute (mL·kg^−1^·min^−1^). Vertical jump was collected as the maximum jump height attained from three trial jumps. The students highest jump measure was recorded in cm from the difference in standing height and maximum jump height.

All data were analyzed using STATA statistical software, version 12.0. Poisson regression analyses controlled for relevant covariates (sex, previous injury, sport participation, intervention group, and aerobic fitness level) and clustering by class was used to estimate the risk of sport injury, across injury definitions, reported using incidence rate ratios (IRR) with 95% confidence intervals (CI). Injury definitions included all sport injury, lower extremity injury, time loss injury, and ankle and knee sprain injury. The covariates considered in the final model were those significant at the 5% level. Two-way interactions were examined for evidence of effect modification by sex, previous injury, and intervention group. In the case of no effect modification, variables were taken out of the model to asses the change in betas to assess for confounding.

## 3. Results

The cohort participants included 1,040 children at the ages of 11–15 years. The median age of participants was 13 years (IQR: 12–13). At baseline (Table 1), the proportion of overweight/obese boys (15.3%, 95% CI: 11.9–18.9) was higher compared to girls (9.0%, 95% CI: 6.7–11.2). In addition, boys had higher levels of aerobic fitness (VO_2_ max estimates = 42.6 mL·kg^−1^·min^−1^, 95% CI: 41.8–43.3) compared to girls (VO_2_ max estimates = 36.8 mL·kg^−1^·min^−1^, 95% CI: 36.3–37.3) in addition to measures of vertical jump (cm) and WC (cm) (Table 1).

When examining the differences in measures of lower extremity strength (vertical jump, measured as the maximum jump height in cm), overweight/obese students had significantly lower measures of vertical jump (29.7 cm, SD = 6.94) compared to healthy weight students [34.0 cm, SD = 7.78, *t*(957) = 5.51, and *p* ≤ 0.0001]. For WC, vertical jump measures were also lower for those students with unhealthy WC (32.1 cm, SD = 7.91) compared to students with healthy measures [34.0 cm, SD = 7.73, *t*(971) = 3.22, and *p* = 0.0013].

There was no evidence of effect modification by sex, previous injury, or intervention type. There were a total of 109 injuries that occurred during the study period, which represents an overall cohort injury rate of 1.80 injuries/1000 total hours of sport participation. The rate of lower extremity injury was 1.27/1000 hours, time loss injury was 0.63/1000 hours, knee injury was 0.48/1000 hours, and ankle injury was 0.45/1000 hours. The Poisson regression model demonstrated an increased risk of time loss injury (IRR = 2.82, 95% CI: 1.01–8.04) and knee injury (IRR = 2.07, 95% CI: 1.00–6.94) in overweight/obese adolescents (Table 2). There were no significant increases in injury risk for all sport injury and lower extremity injury in overweight/obese adolescents. Point estimates suggest a greater risk of time loss injury [IRR = 1.63 (95% CI: 0.93–2.47)] and knee injury [IRR = 1.43 (95% CI: 0.60–3.47)] in adolescents with unhealthy measures of WC; however, these effects were not statistically significant.

## 4. Discussion

This study was a follow-up to previous research demonstrating that obese students were at a 34% increased risk for sport injury, compared to healthy weight students [7]. The results of this study show that overweight/obese children are at a higher risk of time loss injury and knee injury, compared to healthy weight students. Overweight/obese adolescents may be at increased risk for sport-related injury due to the forces being absorbed through soft tissue and joints. Literature in this area has long established that a lack of lower extremity strength and power increases the risk of knee and ankle related injury [15]. There is an increased risk of anterior cruciate ligament injury (greater in females) with greater hamstring : quadriceps ratios [15]. In this study, students with greater unhealthy mass have poorer lower extremity strength that may be related to the lack of exposure to the type of activities that would increase lower extremity strength.

There is a breadth of literature describing components of neuromuscular training programs that reduce the burden of pediatric sport injury in a sport-specific population [16–21]. Less information is known about the components of programs that can reduce injury in the general population (e.g., students) [8, 22]. It is important to consider all potentially modifiable risk factors for sport injury in the development of prevention programs. Including exercises that can reduce measures of BMI as a risk factor of injury and, at the same time, increasing lower extremity strength and balance will have the greatest impact. In addition, applying these programs to children outside of a sport-specific setting can have large public health impact.

Strengths of this study include the prospective collection of sport injury assessed weekly by a study physiotherapist and direct measures of weight and fitness level. This study also prospectively collected hours of sport participation, which was used as the offset to represent a rate of injury per hour of participation. An additional strength of this study includes the low drop-out rate (0.41%) and the potential for socioeconomic status as a confounder was eliminated due to the homogeneity between the two groups being compared. Limitations in this study include the relatively small proportion of the population categorized as being overweight/obese (11.6%, 95% CI: 9.6–13.5). The RCT that these data were initially collected from was powered on an expected difference in injury proportion between the two schools. The ability to evaluate numerous injury definitions across primary exposure variables of interest leads to wide confidence intervals and increased risk of type II error. In addition, it is impossible to consider sport type, mechanism of injury, and injury type in the modeling of these data due to the numerous sports, mechanisms, and injury types reported. This may lead to spurious conclusions of overweight/obesity as a risk factor for sport injury. Upon visual examination of these data, however, the proportion of overweight/obese students participating in contact sports did not appear to be different compared to those of healthy weight. Finally, these data were limited to two schools; therefore, the generalizability of these data to populations beyond those reported here is limited.

## 5. Conclusion

The results from this study suggest that there is an increased risk of sport-related injury in overweight/obese adolescents when considering exposure to participation. Specifically, adolescents with higher BMIs demonstrate an increased risk of more severe injury (time loss greater than seven days) and knee injuries. Sport injury prevention training programs should include strategies targeting both injury prevention and healthy body weight.

## Figures and Tables

**Table 1:** Baseline characteristics by sex.

	Boys	Girls
Frequency, %	427 (41.0)	613 (58.9)
Age (median, IQR)	13 (12-13)	13 (12-13)
BMI (mean, 95% CI)	19.5 (19.2–19.8)	19.3 (19.1–19.5)
Missing (*n*)	9	9
Unhealthy BMI (%, 95% CI)	15.3 (11.9–18.9)	9.0 (6.7–11.2)
WC (mean, 95% CI)	69.2 (68.4–70.1)	66.5 (66.0–67.1)
Missing (*n*)	7	9
Unhealthy WC (%, 95% CI)	24.8 (20.7–28.9)	22.7 (19.4–26.0)
Sport participation^*∗*^ (median, IQR)	52.0 (32.0–80.0)	52.0 (31.0–77.0)
Aerobic fitness^*∗∗*^ (mean, 95% CI)	42.6 (41.8–43.3)	36.8 (36.3–37.3)
Missing (*n*)	11	25
Vertical jump (cm)	36.9 (36.1–37.8)	31.2 (30.7–31.7)
Missing (*n*)	28	39
Previous injury^†^ (%, 95% CI)	23.5 (19.5–27.6)	22.4 (19.1–25.8)
Missing (*n*)	6	7

BMI: body mass index (kg/m^2^). WC: waist circumference (cm).

^*∗*^Total sport participation over study period (hours). ^*∗∗*^Indirect measure of aerobic fitness. ^†^Injury reported in the previous 12 months (frequency).

**Table 2:** Incidence rates (IR, 95% CI) and incidence rate ratios (IRR, 95% CI) by risk factors, adjusted for previous injury, sex, aerobic fitness, intervention group, and sport participation.

	Number of injuries	All sport IR (*n* = 109)	Lower extremity IRR^*∗*^ (*n* = 77)	Time loss IRR^*∗*^ (*n* = 38)	Knee IRR^*∗*^ (*n* = 29)	Ankle IRR^*∗*^ (*n* = 27)
BMI (kg/m^2^)						
Healthy^*∗∗*^	94	1.00	1.00	1.00	1.00	1.00
Unhealthy^*∗∗*^	11	1.28 (0.65–2.49)	1.14 (0.50–2.61)	2.82 (1.01–8.04)	2.07 (1.00–6.94)	0.44 (0.06–3.43)
WC (cm)						
Healthy^*∗∗∗*^	84	1.00	1.00	1.00	1.00	1.00
Unhealthy^*∗∗∗*^	25	1.26 (0.78–2.07)	1.14 (0.90–1.75)	1.63 (0.93–2.47)	1.43 (0.60–3.47)	0.59 (0.17–2.05)
Previous injury (yes/no)						
No	69	1.00	1.00	1.00	1.00	1.00
Yes	38	1.47 (1.00–2.15)	1.43 (0.83–2.59)	2.16 (1.06–4.42)	2.60 (1.00–6.76)	1.25 (0.55–2.85)
Sex (males/females)						
Females	59	1.00	1.00	1.00	1.00	1.00
Males	50	0.78 (0.54–1.12)	0.73 (0.46–1.16)	0.58 (0.26–1.30)	0.54 (0.19–1.52)	0.74 (0.33–1.68)
Intervention (yes/no)						
No	60	1.00	1.00	1.00	1.00	1.00
Yes	49	0.30 (0.18–0.51)	0.30 (0.18–0.52)	0.37 (0.17–0.81)	0.32 (0.11–0.93)	0.37 (0.18–0.77)

BMI: body mass index. WC: waist circumference. ^*∗*^Adjusted model. ^*∗∗*^Based on international cutoffs. ^*∗∗∗*^Based on national reference data.
